# The association between lifestyle risk score and mental health in Iranian overweight and obese women: a cross-sectional study

**DOI:** 10.3389/fnut.2025.1533453

**Published:** 2025-03-28

**Authors:** Sara Ebrahimi, Farideh Shiraseb, Maryam Ladaninezhad, Negin Navaei, Azimeh Izadi, Khadijeh Mirzaei

**Affiliations:** ^1^Institute for Physical Activity and Nutrition (IPAN), School of Exercise and Nutrition Sciences, Deakin University, Geelong, VIC, Australia; ^2^Department of Community Nutrition, School of Nutritional Sciences and Dietetics, Tehran University of Medical Sciences (TUMS), Tehran, Iran; ^3^School of Nutritional Sciences and Dietetics, Tehran University of Medical Sciences, Tehran, Iran; ^4^Department of Nutrition, College of Graduate and Undergraduate Studies, Life University, Marietta, GA, United States; ^5^Department of Medical, Orchid Pharmed Company, Tehran, Iran

**Keywords:** lifestyle, mental health, Iranian women, lifestyle risk score, obesity

## Abstract

**Background:**

Since evidence on the relationship between a combination of lifestyle factors and mental health in the Iranian population is limited, this study employed a cross-sectional design which is a quick and low-cost method to provide more information on the potential association between lifestyle and mental health. This study addresses this gap by focusing on Iranian overweight and obese women.

**Methods:**

This cross-sectional study analyzed 278 Iranian overweight and obese women. A multistage random sampling method was used to recruit the participants. The lifestyle risk score (LRS) was created based on diet, physical activity (PA), sleep, obesity, and sociodemographic characteristics. Multinomial logistic regression analysis was used to evaluate the association between LRS and the odds of depression, anxiety, and stress. Participants were assigned a score of 0 for each healthy behavior and a score of 1 for each unhealthy behavior. A higher LRS indicates an unhealthier lifestyle. A binary logistic regression analysis was used to examine LRS and the stress and depression anxiety stress scale (DASS).

**Results:**

Significant positive associations between high LRS and moderate and severe depression were found (*p* < 0.05). Furthermore, there were significant positive associations between higher LRS and mild and extremely severe stress (*p* < 0.05).

**Conclusion:**

This is the first study that examined associations between LRS and total DASS-21 and demonstrated that participants with lower LRS had lower levels of depression and stress. This study highlights the crucial role of healthy lifestyle choices in psychological wellbeing. These findings inform the design of interventions to address mental health disorders in Iran. Further prospective studies, including a larger sample size of both genders, are needed to expand our understanding of lifestyle scores’ associations with mental health.

## Background

The World Health Organization (WHO) recognizes mental health as a crucial component of overall wellbeing where people effectively manage typical life pressures, engage in productive work, and actively contribute to their community (1). There has been a 13% increase in the prevalence of mental health worldwide over the past decade. These disorders account for 20% of the years lived with disability (2). In line with global trends, mental disorders such as depression and anxiety are also widespread in the Eastern Mediterranean Region (EMR), with a higher prevalence in women than men (3). A systematic review and meta-analysis reported that the prevalence of depression was 34% in 2020 in the Iranian population (4).

Mental health is linked to different lifestyle factors and health behaviors (5, 6). The evidence showed a two-way association between obesity and mental disorders, where obese people are 55% more likely to experience depression, and individuals with depression have a 58% higher chance of developing obesity (7–9). Also, abdominal obesity was reported to be associated with anxiety in Iranian females (10). Furthermore, a previous study reported the association between cardiovascular health and depression (11). A healthier diet with a higher consumption of fruits, vegetables, nuts, and legumes and a lower intake of red meat, is linked to a lower likelihood of experiencing cardiovascular diseases and depression (12). Physical activity (PA) interventions reduced the symptoms of anxiety and depression (13, 14). Sleep disruptions are likely to play a contributing role in the development of various mental disorders (15). Lower socioeconomic status (SES) is associated with a higher prevalence of depression (16).

The association between mental health and obesity (10), PA (17), sleep quality (18), dietary pattern (19), and SES (20) have been previously investigated in the Iranian population. These studies focused on a single factor in relation to mental health. However, a combination of various factors may simultaneously alter mental health. Lifestyle scores assess multiple factors in relation to health outcomes (21, 22). A previous study on 28,138 Chinese adults showed negative associations between lifestyle score and depression and anxiety (5). The lifestyle score used in this study included smoking, drinking, diet behaviors, PA, sitting time, sleep duration, and sleep quality (5). Despite growing evidence globally, studies on the association between lifestyle scores and mental health in Iranian adults remain scarce. Only one study examined associations between lifestyle score and mental health, which included adults from Isfahan, Iran, and assessed depression and anxiety. As a result, this study aimed to investigate the associations between the LRS and depression, anxiety, and Depression Anxiety Stress Scales (DASS) in overweight and obese Iranian women. By addressing multiple lifestyle factors simultaneously, this study seeks to provide a more comprehensive understanding of how lifestyle behaviors collectively influence mental health in the Iranian population.

## Methods

### Study population

This cross-sectional study involved 278 overweight and obese Iranian women aged ≥18 years, with body mass index (BMI) ≥ 25 kg/m^2^. Participants were recruited through a multistage random sampling method from the community health centers affiliated with the Tehran University of Medical Sciences. Women were excluded if they had cardiovascular diseases, stroke, kidney disorders, liver diseases, thyroid disorders, inflammatory conditions, cancer, pregnancy, lactation, or menopause. Additional exclusion criteria included extreme dietary energy intake (<800 or > 4,200 kcal/day), ongoing weight loss programs or taking weight loss medications and failure to respond to more than 70 questions in the food frequency questionnaire (FFQ). A trained nutritionist conducted all the face to face interviews. The study was approved by the Tehran University of Medical Sciences Ethics Committee (ethics number: IR.TUMS.VCR.REC.1398.142) (23, 24).

### Sociodemographic characteristics and anthropometric indices

Sociodemographic data—including age, education level, marital status, occupation, and income—were collected using a structured questionnaire. Educational levels were categorized into high (bachelor’s degree or higher) and low (diploma or lower), while employment status was classified as employed or non-employed. Income was categorized as high (above the poverty line) or low (below the poverty line), with the poverty line defined as 11.5 million rials per person in 2018. Socioeconomic status (SES) was determined based on occupation, education, and income, similar to prior research. Participants were categorized into high SES (SES score ≥ 2) and low SES (SES score < 2) (25).

Anthropometric measurements were performed with participants in light clothing and without shoes. Body weight was measured to the nearest 0.1 kg using a Seca digital scale (Germany), and height was measured to the nearest 0.2 cm using a Seca 206 stadiometer (Germany). Waist circumference (WC) and hip circumference (HC) were measured to the nearest 0.2 cm using a flexible tape measure. Body composition, including fat-free mass (FFM) and body fat mass (BFM), was assessed using an InBody770 scanner. Waist-to-height ratio (WHtR) was calculated as WC (cm) divided by height (cm), with participants classified as non-obese (WHtR ≤0.5) or obese (WHtR >0.5). WHtR is considered an early indicator of health risks associated with central obesity (26).

### Biochemical parameters

Participants underwent fasting blood tests after 10–12 h of overnight fasting at the Tehran University of Medical Sciences’ nutrition laboratory. Fasting blood sugar (FBS) was measured using the Glucose Oxidase Phenol 4-Aminoantipyrine Peroxidase method, while serum insulin was measured using a radio-immune assay. Aspartate transaminase (AST) and alanine transaminase (ALT) levels were assessed using the International Federation of Clinical Chemistry and Laboratory methods. Insulin resistance (IR) was calculated using the Homeostatic Model Assessment for Insulin Resistance (HOMA-IR) formula: fasting insulin (mIU/L) × FBS (mmol/L)/22.5. Lipid profiles—including triglycerides (TG), total cholesterol (TC), high-density lipoprotein (HDL), and low-density lipoprotein (LDL)—were measured using enzymatic methods (Pars Azmun Co., Tehran, Iran).

### Dietary intake assessment

Dietary intake was assessed using a validated 147-item semi-quantitative FFQ (27). Participants were asked about the frequency of consumption of various foods over the past year, with trained dietitians conducting face-to-face interviews. Nutrient and energy intakes were analyzed using the NUTRITIONIST-IV software (version 7.0; N Squared Computing, Salem, OR, United States). Given the link between cardiovascular and mental health, dietary behaviors were assessed using the American Heart Association (AHA) diet score, which was based on FFQ data and developed according to AHA guidelines (6, 28). The AHA diet score included eight components: fruits and vegetables, fish and shellfish, sodium, sugar-sweetened beverages, whole grains, nuts and legumes, processed meats, and saturated fats. Each component was scored from 0 to 10, with a total possible score ranging from 0 to 80. Participants were categorized as having low adherence (AHA score < 40) or high adherence (AHA score ≥ 40) to dietary recommendations (29–31) (Supplementary Table 1).

### Sleep quality and PA assessment

Participants’ sleep quality was evaluated using the Pittsburgh Sleep Quality Index (PSQI) which includes subjective sleep quality, sleep latency, sleep duration, habitual sleep efficiency, sleep disturbances, use of sleep medications, and daytime dysfunction. The PSQI score range is between 0 and 21, and a total score over five implies poor sleep quality (32). PA was assessed using the International Physical Activity Questionnaire (IPAQ) questionnaire (33). Participants were categorized into moderate or high levels of PA (> 20 MET-h/week) and low levels of PA (≤ 20 MET-h/week).

### Mental health assessment

Individual’s mental health was evaluated using DASS-21. DASS-21 comprises 21 items over three stages. Each stage assesses seven items with a range of scores between 0 and 3. A higher score indicates poorer mental health (23, 34).

### LRS assessment

The LRS was calculated based on five lifestyle factors: sleep quality, SES, WHtR, dietary behavior, and PA. Participants were assigned a score of 0 for each healthy behavior (AHA score ≥ 40, PA >20 MET-h/week, PSQI <5, WHtR <0.5, SES ≥2) and a score of 1 for each unhealthy behavior. Participants with a total LRS above the median (score > 2) were classified as the high-risk group, while those with an LRS ≤2 were categorized as the low-risk group. A higher LRS indicates an unhealthier lifestyle (Supplementary Table 2).

### Statistical analysis

The data were analyzed using SPSS software (version 21). The Kolmogorov–Smirnov test was employed to assess the normality of dependent variables, with a *p*-value >0.05 indicating normal distribution. Continuous variables were reported as means and standard deviations (SD), while categorical variables were presented as frequencies and percentages. Group comparisons of continuous variables were performed using a one-way analysis of variance (ANOVA). Categorical variables were compared between groups using the chi-square or Fisher’s exact test. For adjusted analyses, analysis of covariance (ANCOVA) was used, controlling for potential confounders such as age and energy intake. BMI, PA, and educational status were treated as collinear variables. Multinomial logistic regression was conducted to assess the association between LRS and the odds of depression, anxiety, and stress. In addition, binary logistic regression was used to explore the relationship between LRS and DASS. Two adjustment models were created: Model 1 adjusted for age and energy intake, while model 2 included further adjustments for economic status, supplement use, and marital status. A *p*-value <0.05 was considered statistically significant, while *p*-values of 0.06, 0.07, and 0.08 were interpreted as marginally significant.

## Results

### Sociodemographic characteristics of the study participants

Participants’ mean (SD) of age, weight, BMI and WC was 36.5 (8.53) years, 80.7 (12.37) kg, 31.1 (4.39) kg/m^2^, and 95.5 (16.13) cm, respectively. About 49.5% of participants had a higher level of education, while only 12.5% had an education level under a diploma. The majority of participants (74%) were married, had high LRS (66%). In the present study, 10.4 and 6.5% of participants had severe and extremely severe depression, respectively, while 17.6, 13.3, and 6.5% had moderate, severe, and extremely severe stress, respectively. Furthermore, 24.5 and 14.7% of participants experienced moderate anxiety and extremely severe anxiety, respectively.

### Sociodemographic characteristics of participants over categories of LRS

The sociodemographic characteristics of participants over the categories of LRS are presented in Table 1. Individuals with higher LRS had higher weight (*p* = 0.007), higher BMI (*p* < 0.001), higher body fat (*p* < 0.001), and lower PA (*p* = 0.017). There was a marginally significant mean difference in age (*p* = 0.070), height (*p* = 0.065), and LDL cholesterol level (*p* = 0.005) between low and high LRS groups in the crude model before adjustment. However, the difference disappeared after controlling for age and energy intake.

**Table 1:** Characteristics of participants over the LRS categories (*n* = 278).

Variables	LRS		*P*-value	*P*-value*
	Low risk	High risk		
	≤2 score	>2 score		
	Mean ± SD			
Continuous variables				
Age (year)	34.441 ± 8.037	36.798 ± 9.018	**0.070**	0.133
Weight (Kg)	78.139 ± 13.361	81.434 ± 11.563	**0.069**	**0.007**
Height (cm)	162.144 ± 5.627	160.520 ± 6.050	**0.065**	0.222
PA (MET min/ week)	1276.1417 ± 1233.660	783.905 ± 915.377	**0.003**	**0.017**
BMI (Kg/m^2^)	29.563 ± 4.264	31.593 ± 4.090	**0.001**	**< 0.001**
WC (cm)	92.543 ± 22.413	95.726 ± 9.199	0.294	0.185
WHtR (cm)	0.913 ± 0.0607	1.612 ± 7.837	0.460	0.396
Body fat mass (Kg)	31.442 ± 8.691	34.916 ± 8.351	**0.006**	**< 0.001**
FBS (mg/L)	85.793 ± 8.625	88.196 ± 9.927	0.109	0.612
TG (mg/L)	115.661 ± 65.789	124.288 ± 58.766	0.376	0.843
Chol (mg/L)	175.396 ± 28.874	184.214 ± 33.883	0.084	0.449
HDL (mg/L)	49.238 ± 10.402	47.125 ± 10.356	0.198	0.295
LDL (mg/L)	91.793 ± 19.447	101.455 ± 22.840	**0.005**	0.096
Insulin (mIU/mL)	1.214 ± 0.252	1.235 ± 0.226	0.566	0.838
HOMA_IR index	3.150 ± 1.393	3.472 ± 1.365	0.146	0.261
Categorical variables n (%)				
Supplementation intake (%)				
Yes %	36 (32.4)	75 (67.6)	0.896	0.831
No %	27 (33.3)	54 (66.7)		
Educational status (%)				
Diploma and under-diploma	37 (35.4)	66 (64.6)	0.910	0.328
Bachelor and higher	31 (31.3)	68 (68.7)		
Marital status (%)				
Single	20 (34.5)	38 (65.5)	0.876	0.911
Married	48 (33.3)	96 (66.7)		
Economic status (%)				
Poor	17 (33.3)	34 (66.7)	0.178	0.284
Moderate	28 (29.5)	67 (70.5)		
Good	24 (44.4)	30 (55.6)		

PA, physical activity; BMI, body mass index; WC, waist circumstance; WHtR, waist to hip ratio; FBS, fasting blood sugar; TG, Triglycerides; chol, Cholesterol; LDL, Low-density lipoprotein cholesterol; LRS, Lifestyle risk score; HDL, High-density lipoprotein cholesterol; HOMA-IR index, Homeostatic Model Assessment of Insulin Resistance index. Means ± SD, and number (%) were presented for continuous and categorical variables, respectively. *P-value was driven using ANCOVA. The analysis was adjusted for age, and energy intake. *p*-value < 0.05 was considered significant and *p*-values of 0.05, 0.06, and 0.07 were considered marginally significant. The bold values are statistically significant and statistically marginally significant.

### Dietary intakes over the categories of LRS

The dietary intakes of participants across the median of LRS are shown in Table 2. After controlling for energy, it was found that participants with low LRS had a higher intake of trans fatty acid, which was marginally significant (*p* = 0.077). In addition, women with lower LRS had a higher intake of vegetables (*p* = 0.009), whole grains (*p* = 0.001), nuts (*p* = 0.062), and legumes (*p* < 0.001). The intake of vitamin A (*p* = 0.073), and vitamin B6 (*p* = 0.075) were marginally significantly different between LRS groups where women with lower LRS had a higher intake.

**Table 2:** Participant’s dietary intake over LRS categories (*n* = 278).

Variables	LRS		*P*-value	*P*-value*
	Low risk	High risk		
	≤2 score	>2 score		
	Mean ± SD			
Energy and macronutrients				
Energy intake (kcal/d)	2683.504 ± 769.782	2609.936 ± 735.242	0.507	–
CHO (% TEI)	57.189 ± 6.080	56.891 ± 6.466	0.755	0.661
Fat (% TEI)	31.491 ± 6.074	32.244 ± 5.839	0.399	0.339
Protein (% TEI)	14.308 ± 2.389	13.853 ± 2.523	0.224	0.231
SFA (mg/d)	28.016 ± 10.954	28.998 ± 11.972	0.569	0.235
PUFA (mg/d)	19.707 ± 9.313	20.535 ± 9.282	0.548	0.376
MUFA (mg/d)	30.731 ± 11.611	32.178 ± 12.259	0.418	0.158
Trans fatty acid (g/d)	0.001 ± 0.003	0.000 ± 0.001	0.095	**0.077**
Total dietary fiber (g/d)	45.918 ± 18.794	45.179 ± 18.585	0.789	0.175
Linoleic Acid (g/d)	16.763 ± 8.899	17.780 ± 8.780	0.437	0.324
Linolenic Acid (g/d)	1.312 ± 0.667	1.287 ± 0.730	0.813	0.853
EPA (g/d)	0.037 ± 0.040	0.029 ± 0.034	0.161	0.298
DHA (g/d)	0.121 ± 0.128	0.977 ± 0.105	0.166	0.299
Micronutrients				
Vit A (mg/d)	854.962 ± 459.461	739.181 ± 363.656	**0.051**	**0.073**
Vit C (mg/d)	195.145 ± 115.156	195.739 ± 137.680	0.975	0.630
Vit E (mg/l)	16.227 ± 8.645	17.441 ± 9.076	0.359	0.410
Ca (mg/d)	1209.352 ± 491.846	1155.937 ± 402.943	0.408	0.645
Iron (mg/d)	19.726 ± 6.295	18.405 ± 5.747	0.134	0.177
Thiamin (mg/d)	2.129 ± 0.686	2.090 ± 0.638	0.359	0.686
Niacin (mg/d)	26.359 ± 9.213	25.214 ± 9.864	0.424	0.940
Riboflavin (mg/d)	2.253 ± 0.878	2.191 ± 0.850	0.631	0.915
Vit B5 (mg/d)	6.969 ± 2.369	6.406 ± 2.672	0.141	0.252
Vit B6 (mg/d)	2.311 ± 0.771	2.107 ± 0.703	**0.060**	**0.075**
Biotin (mg/d)	41.570 ± 15.831	36.700 ± 18.738	0.066	0.178
Folate (mcg/d)	636.654 ± 187.012	595.279 ± 170.730	0.114	0.229
Vit B12 (mcg/d)	4.791 ± 3.498	4.209 ± 1.998	0.132	0.162
Zinc (mg/d)	13.841 ± 4.397	12.589 ± 4.143	0.470	**0.022**
Copper (mg/d)	2.127 ± 0.715	1.969 ± 0.741	0.147	0.262
Selenium (mg/d)	123.253 ± 41.609	118.533 ± 42.388	0.450	0.908
Chromium (mg/d)	0.119 ± 0.083	0.106 ± 0.081	0.281	0.512
Caffeine (mg/d)	124.264 ± 84.883	140.556 ± 98.421	0.244	0.175
Food groups				
Fruits (g/d)	545.875 ± 341.252	502.376 ± 329.766	0.362	0.470
Vegetables (g/d)	501.574 ± 289.534	403.485 ± 234.536	**0.008**	**0.009**
Whole grains (g/d)	9.830 ± 11.939	5.314 ± 7.586	**0.001**	**0.001**
Nuts (g/d)	17.026 ± 16.918	12.727 ± 14.690	**0.053**	**0.062**
Legumes (g/d)	67.270 ± 51.587	45.918 ± 33.616	**< 0.001**	**< 0.001**
Tea and coffee (g/d)	615.979 ± 407.130	684.963 ± 491.668	0.299	0.203
Refined grains (g/d)	443.446 ± 245.393	429.255 ± 192.552	0.640	0.670
Dairy (g/d)	418.343 ± 273.859	382.369 ± 226.740	0.304	0.365
Red meat (g/d)	25.113 ± 19.926	19.982 ± 16.767	0.046	0.053
White meat (g/d)	51.728 ± 45.176	45.398 ± 49.972	0.360	0.399
Processed food (g/d)	21.445 ± 18.627	25.790 ± 30.846	0.265	0.207

CHO, Carbohydrate; DHA, Docosahexaenoic acid; EPA, eicosatetraenoic acid; LRS, Lifestyle risk score, MUFA, monounsaturated fatty acid; SFA, saturated fatty acid; Pro, protein; PUFA, polyunsaturated fatty acid; TEI, Total energy intake. Values are represented as means (SD). *P-value was driven using the ANCOVA test. The analysis was adjusted for energy intake by ANCOVA test. *p*-value < 0.05 was considered significant and *p*-values of 0.05–0.07 were considered marginally significant. The bold values are statistically significant and statistically marginally significant.

### Associations between LRS and depression, anxiety, stress, and total DASS-21

The association between LRS and total DASS-21 and its subscales including depression, anxiety, and stress are shown in Table 3. Before and after adjustment for confounders, significant positive associations between high LRS and moderate (Crude model: OR: 3.0, CI: 1.3, 7.2; *p*-value: 0.012; model 1: OR:2.8, CI: 1.1, 7.1; *p*-value: 0.031; model 2: OR: 2.8, CI: 1.1, 7.7; *p*-value: 0.030) and severe depression (Crude model: OR: 3.1, CI: 0.98, 9.9; *p*-value: 0.014; model 1: OR: 2.5, CI: 1.8, 8.3; *p*-value: 0.046; model 2: OR: 3.2, CI: 1.9, 11.7; *p*-value: 0.049) were found. Furthermore, before and after adjustment for confounders, there were significant positive associations between higher LRS and mild stress (Crude model: OR: 1.4, CI: 1.2, 4.0; *p*-value: 0.017; model 1: OR:1.4, CI: 1.2, 4.9; *p*-value: 0.040; model 2: OR: 1.4, CI: 1.2, 4.1; *p*-value: 0.048) and extremely severe stress (Crude model: OR: 3.0, CI: 1.6, 14.4; *p*-value: 0.010; model 1: OR:2.8, CI: 1.6, 9.6; *p*-value: 0.021; model 2: OR: 2.5, CI: 5.0, 10.5; *p*-value: 0.044). Also, there was a marginally significant positive association between higher LRS and total DASS-21 before and after adjustment for confounders (Crude model: OR: 1.4, CI: 0.79, 2.5; *p*-value: 0.060; model 1: OR: 1.4, CI: 0.75, 2.6; *p*-value: 0.074; model 2: OR: 1.4, CI: 0.74, 2.7; *p*-value: 0.075).

**Table 3:** Associations between mental health and LRS (*n* = 278).

Variables	High LRS								
	Crude model			Model 1			Model 2		
	OR	95% CI	*p*-value	OR	95% CI	*P*-value	OR	95% CI	*P*-value
Depression									
Mild	1.742	0.699, 4.340	0.234	1.784	0.695, 4.580	0.229	1.280	0.443, 3.698	0.648
Moderate	3.025	1.274, 7.183	**0.012**	2.789	1.100, 7.070	**0.031**	2.777	1.102, 7.696	**0.030**
Severe	3.117	0.980, 9.908	**0.014**	2.530	1.769, 8.318	**0.046**	3.199	1.875, 11.696	**0.049**
Extremely severe	0.880	0.252, 3.078	0.841	1.014	0.277, 3.708	0.983	1.344	0.336, 5.382	0.676
Anxiety									
Mild	1.765	0.588, 5.304	0.311	2.132	0.670, 6.781	0.200	2.014	0.576, 7.041	0.273
Moderate	0.923	0.440, 1.937	0.832	0.832	0.375, 1.844	0.651	0.929	0.380, 2.274	0.873
Severe	0.802	0.330, 1.953	0.628	0.870	0.335, 2.260	0.775	0.867	0.302, 2.487	0.790
Extremely severe	1.103	0.422, 2.883	0.841	0.776	0.279, 2.152	0.626	1.028	0.326, 3.240	0.962
Stress									
Mild	1.440	1.189, 4.025	**0.017**	1.386	1.156, 4.958	**0.040**	1.416	0.161, 4.076	**0.048**
Moderate	1.775	0.733, 4.301	0.204	1.546	0.620, 3.856	0.350	1.394	0.549, 3.543	0.485
Severe	0.733	0.286, 1.880	0.518	1.691	0.252, 1.895	0.472	1.677	0.243, 1.887	0.455
Extremely severe	3.043	1.645, 14.357	**0.010**	2.800	1.577, 9.584	**0.021**	2.500	0.501, 10.470	**0.044**
Total DASS-21	1.412	0.792, 2.513	**0.060**	1.413	0.750, 2.662	**0.074**	1.425	0.744, 2.732	**0.075**

OR, odd ratio; CI, Confidence Interval; DASS, depression anxiety stress score; LRS, Lifestyle risk score. Model 1: The analysis was adjusted for age, energy intake. Model 2: The analysis was adjusted for age, energy intake, economic situation, supplement consumption, and marital status. The normal group was considered the references group. A group lower than the median for DASS-21 was considered a reference group. The odds ratio (OR) has been reported. Multinomial logistic regression was used to examine associations between depression, anxiety, stress and LRS, while a binary logistic regression analysis was used to test associations between DASS-21 and LRS. A high lifestyle risk score is more than the median (>2 score) *P*-value < 0.05 was considered significant, and *p*-values of 0.05–0.07 were considered marginally significant. The bold values are statistically significant and statistically marginally significant.

## Discussion

This study examined the associations between the LRS and mental health in overweight and obese Iranian women. The results revealed that a higher LRS was positively correlated with moderate to severe depression and mild to extremely severe stress, with no significant association found between the LRS and anxiety. Additionally, the overall DASS-21 score exhibited a marginal association with higher LRS values.

This study found a positive association between the LRS and moderate and severe depression. In line with our findings, a study on 28,138 Chinese adults reported a negative association between a healthier lifestyle and depression. The lifestyle factors included smoking, drinking, diet behaviors, PA, sitting time, sleep duration, and sleep quality (5). Furthermore, another study on 6,054 Belgian adults found a negative association between a healthier lifestyle and depression. The lifestyle score comprised BMI, smoking status, PA, alcohol consumption and diet. A study on 3,363 Iranian adults from Isfahan examined associations between lifestyle score and depression and demonstrated that participants with a higher score of healthier lifestyles were less likely to have depression. This study considered dietary intake, PA, smoking status, psychological distress and central obesity to develop the lifestyle score (35). These findings might be attributed to dietary intake, where participants with lower LRS had a higher intake of vegetables, whole grains, nuts and legumes. The evidence indicates that fruits and vegetables are sources of nutrients including fiber, vitamin C, B vitamin and polyphenols which are recognized for their potential relationship with mental health (36). Furthermore, diets with a high glycaemic index and glycaemic load, such as those rich in refined carbohydrates and sugars, may negatively impact psychological wellbeing through rapid increases and decreases in blood glucose (37, 38). A high dietary glycemic load may reduce plasma glucose to levels that stimulate the release of autonomic counter-regulatory hormones, such as cortisol, adrenaline, growth hormone, and glucagon. These findings suggest that counterregulatory hormones may contribute to changes in anxiety, irritability, and hunger (39–41). Furthermore, the Mediterranean diet, characterized by a higher intake of fruits and vegetables, cereals, nuts and legumes, has been reported to be associated with lower inflammatory markers and fewer depressive symptoms (42). The evidence shows that inflammatory mechanisms may develop depression through hindering neurotransmitter metabolism and initiating endothelial dysfunction (43, 44). On the other hand, an unhealthy diet with a high intake of energy and saturated fat may decline cognitive and hippocampal function and impair the blood–brain barriers (42). This suggests adherence to a healthier diet and whole foods may reduce the probability of depression development (44, 45). In addition, participants with lower LRS had higher levels of PA. It is well established that PA is a key intervention to boost mental health (46). The European Psychiatry Association and the International Organization of Physical Therapists in Mental Health have released a statement regarding the utilization of PA in the management of mental disorders (47, 48). PA may promote mental health through neuroendocrine and inflammatory responses to activity, such as the activation of the endocannabinoid system, as well as long-term adaptations, including alterations in the brain’s neural structure. Furthermore, psychosocial and behavioral factors have also been proposed, such as enhanced physical self-perception and body image, increased social interactions, and the development of personal coping strategies (48–52).

Participants with higher LRS also had higher weight, BMI and body fat. While there is conflict in the evidence on the association between obesity and mental health, two systematic reviews and meta-analyses concluded a bidirectional relationship between depression and obesity (9, 53). It might be attributed to the fact that individuals who suffer from depression have a high intake of food and lower PA levels which are the causes of obesity. Also, the negative body image of obese people might aggravate their mental health (54).

While this study found no association between the LRS and anxiety, two previous studies on 3,363 Iranian adults and 28,138 Chinese adults found negative associations between a healthier lifestyle and anxiety. The discrepancies might be attributed to different sample sizes, characteristics of participants, and lifestyle factors used in these studies. While our study included 278 overweight and obese Iranian women, Saneei et al. included 3,363 Iranian adults and Wang et al. examined 28,138 Chinese adults (5, 35). Furthermore, Saneei et al. included smoking status and psychological distress and Wang et al. comprised smoking, drinking, and sitting time to develop the lifestyle score (5, 35). A healthier lifestyle might be related to lower anxiety through oxidative stress pathways. The evidence showed individuals with anxiety have higher levels of inflammation which could be decreased through healthier lifestyles such as healthier diet, and higher PA (55–59). Further studies are suggested to investigate lifestyle score’s relationship with anxiety.

The findings of this study demonstrated a positive link between LRS and stress. The evidence on the associations between lifestyle score and stress is limited. While no previous study assessed the link between lifestyle score and stress in the Iranian population, studies on 28,138 Chinese adults and 6,054 Belgian adults revealed that healthier lifestyles were associated with lower perceived pressure and psychological distress, respectively (5, 60). In the present study, participants with lower LRS had a higher intake of healthier foods. The evidence suggests an association between higher adherence to an unhealthier diet and higher levels of stress (5, 61). Furthermore, individuals with higher stress tend to be engaged in unhealthy lifestyle behaviors such as avoiding PA, and decreased sleep duration (5, 62, 63). Given that few studies have investigated the link between lifestyle score and stress, further surveys are needed to provide further evidence in this area.

To the best of our knowledge, the present study was the first to investigate associations between total DASS-21 and LRS. The findings revealed a marginally significant positive association between total DASS-21 and LRS. Consistent with our findings, a study on 7,937 participants from Germany reported an inverse association between a healthier lifestyle score and the score of DASS stress, depression and anxiety. However, this study did not examine the link between total DASS-21 and lifestyle score (64). A study on 239 university students in Australia assessed associations between single lifestyle factors and DASS-21 and concluded that higher quality of diet and sleep, and moderate-vigorous PA were negatively associated with total DASS-21 (65). Given that evidence on the association between lifestyle score and DASS-21 is scant, further studies are needed to provide extensive information to expand our understanding of lifestyle’s role in mental health development.

Several limitations should be considered in the interpretation of the findings of this study. Firstly, the cross-sectional study design is susceptible to reverse causality while examining associations between LRS and DASS-21. As a result, longitudinal studies are required to establish cause and effect relationships and better understand causal pathways. Secondly, this study collected self-reported data on sleeping and physical activity which may have resulted in responses influenced by social desirability. Future studies could improve the information using wearable device data, which would provide more precise and continuous measurements. Furthermore, this study collected dietary data using the FFQ which is prone to memory bias. Thirdly, this study only included overweight and obese women from Tehran, which is not a representative sample of the Iranian population and restricts the generalizability of these findings to Iranians from diverse socioeconomic backgrounds and regions of Iran. Fourthly, this survey lacked information on alcohol consumption, a common limitation observed in studies from Iran and other Middle Eastern countries (66, 67). Furthermore, there was a lack of information on smoking in this survey. As a result, the LRS created in this study did not contain smoking and alcohol consumption which are associated with mental health. This limitation needs to be considered when comparing results with studies that assessed LRS containing smoking and alcohol consumption. In addition, the LRS total score was categorized based on the median value which may limit the relationship between mental health and lifestyle factors. Fifthly, the small sample size of this study may limit the power to conduct detailed subgroup analyses or explore interactions between variables. Lastly, while this study controlled for several confounding factors, residual confounding may exist.

This study has several strengths. To the best of our knowledge, this study assessed associations between LRS and total DASS-21 for the first time. The analysis was controlled for extensive confounding factors. Furthermore, this study included overweight and obese women who are at higher risk of mental health.

## Conclusion

This study for the first time, examined associations between LRS and total DASS-21 and demonstrated that participants with lower LRS had lower levels of depression and stress. Furthermore, this study revealed a marginally significant positive association between total DASS-21 and LRS. Considering the limitations of this study, future research should aim to address these limitations by including more diverse populations, employing longitudinal designs, and enhancing data collection methods through objective and validated measures.

## Data Availability

The raw data supporting the conclusions of this article will be made available by the authors, without undue reservation.

## Glossary

AHA: American Heart Association
ALT: Alanine transaminase
AST: Aspartate transaminase
ANOVA: one-way analysis of variance
ANCOVA: Analysis of covariance
BMI: body mass index
BFM: body fat mass
DASS: depression anxiety stress scale
EMR: Eastern Mediterranean Region
FBS: fasting blood sugar
FFQ: food frequency questionnaire
FFM: fat-free mass
HDL: high-density lipoprotein
HC: hip circumference
HOMA-IR: Homeostatic Model Assessment-Insulin Resistance
IR: insulin resistance
IPAQ: International Physical Activity Questionnaire
LDL: low-density lipoprotein
LRS: lifestyle risk score
PA: Physical activity
PSQI: Pittsburgh Sleep Quality Index
SD: standard deviation
SES: socioeconomic status
TC: total cholesterol
TG: triglyceride
WC: Waist circumference
WHO: World Health Organization
WHtR: Waist-to-height ratio
