# Supplementary material for: The association between lifestyle risk score and mental health in Iranian overweight and obese women: a cross-sectional study
Source: Front Nutr. 2025 Mar 28;12:1533453. doi: 10.3389/fnut.2025.1533453 (PMC11987328; doi:10.3389/fnut.2025.1533453)
Supplement: Supplementary file 1 [file Table_1.docx]

**Supplementary Table 1**. The American Heart Association components and standards for scoring

| **Components** | **Criteria for scoring** | **Score range** |
| --- | --- | --- |
| Fruits and vegetables | 0 to ≥4.5 cups/d | 0–10 |
| Fish and shellfish | 0 to ≥7 oz/wk | 0–10 |
| Sodium | ≤1500 to >4500 mg/d | 10–0 |
| sugar-sweetened beverages | ≤36 to >210 fl oz/wk | 10–0 |
| Whole grains | 0 to ≥3 oz/d | 0–10 |
| Nuts, seeds, and legumes | 0 to ≥4 servings/d | 0–10 |
| Processed meats | ≤3.5 to >17.5 oz/wk | 10–0 |
| Saturated fat | ≤7 to >15 (% energy) | 10–0 |

**Supplementary Table 2**. The lifestyle risk score components and standards for scoring

| **LRS components** | **Criteria for scoring** | | | **score** |
| --- | --- | --- | --- | --- |
| Dietary behavior |  | | |  |
|  | AHA diet score ≥ 40 | | | 0 |
|  | AHA diet score < 40 | | | 1 |
| PA )MET-h/week( |  | | |  |
|  | moderate to high PA (> 20 MET-h/week) | | | 0 |
|  | low PA (≤ 20 MET-h/week) | | | 1 |
| Sleep Quality |  | | |  |
|  | PSQI ≤ 5 | | | 0 |
|  | PSQI > 5 | | | 1 |
| WHtR |  | | |  |
|  | Non overweight and obese (<0.57) | | | 0 |
|  | Overweright and obese (>0.57) | | | 1 |
| SES |  | | |  |
| Education* | High educational levels | 1 | High SES  (score ≥2) | 0 |
|  | Low educational levels | 0 |  |  |
| Occupation | Employed | 1 |  |  |
|  | Unemployed | 0 | Low SES  (score < 2) | 1 |
| Income** | High income | 1 |  |  |
|  | Low income | 0 |  |  |
| AHA: American Heart Association; LRS: Lifestyle risk score; PA: Physical Activity; PSQI: Pittsburgh sleep quality index; SES: Socioeconomic status; WHtR: Waist to hip ratio  *: Participants were categorized into high education level (bachelor degree and higher), low education level (diploma and lower).  **: High income was considered above poverty line income; low income was considered below poverty line income (eleven million and five hundred thousand rials for each person was considered as poverty line in 2018). | | | | |
